# Presence and molecular characterization of *Clostridium difficile* and *Clostridium perfringens* in intestinal compartments of healthy horses

**DOI:** 10.1186/1746-6148-8-94

**Published:** 2012-06-29

**Authors:** Angelika Schoster, Luis Guillermo Arroyo, Henry Rolf Staempfli, Patricia Elisabeth Shewen, Jeffrey Scott Weese

**Affiliations:** 1 Department of Clinical Studies, Ontario Veterinary College, University of Guelph, Guelph, Canada; 2 Department of Pathobiology, Ontario Veterinary College, University of Guelph, Guelph, Canada; 3 Department of Veterinary Disease Biology, University of Copenhagen, Copenhagen, Denmark

**Keywords:** *Clostridium difficile*, *Clostridium perfringens*, Equine gastrointestinal tract, Equine intestinal microflora, *Clostridium difficile* ribotyping

## Abstract

**Background:**

*Clostridium difficile* and *Clostridium perfringens* are commonly associated with colitis in equids, but healthy carriers exist. *S*carce information is available on the prevalence of *Clostridium* spp. in gastrointestinal compartments other than faeces in healthy horses, and it is unknown whether faecal samples are representative of proximal compartments. The objectives were to investigate the prevalence of *C. difficile* and *C. perfringens* in different intestinal compartments of healthy adult horses and to determine whether faecal samples are representative of colonization in proximal sites and overall carrier status.

**Results:**

Toxigenic *C. difficile* was isolated from 14/135 (10.3%) samples from 8/15 (53.3%) horses. Between zero and three sites were positive per horse, and multiple sites were positive in four horses. Isolates were recovered from duodenum, jejunum, ileum, right dorsal colon, small colon and rectum. When multiple compartments were positive in a single horse, two different *C. difficile* ribotypes were always present. *Clostridium perfringens* Type A (CPE, β_2_ toxin gene negative) was recovered from the left ventral colon of one horse (0.74%, 1/135 samples). Agreement between faeces and overall *C. difficile* carrier status was good.

**Conclusions:**

*Clostridium difficile* can be found in different compartments of the gastrointestinal tract of healthy horses, and multiple strains can be present in an individual horse. The prevalence of *C. perfringens* in healthy adult hoses was low, consistent with previous reports. Faecal samples were representative for presence of *C. difficil*e in proximal compartments in 5/8 horses (63%) but were not representative for the specific strain.

## Background

Over the past decade *Clostridium difficile* has emerged as an important enteric pathogen in human [1] and veterinary medicine [2]. *Clostridium difficile* is an important cause of acute enterocolitis in horses [3-5] with clinical signs ranging from mild self-limiting disease to fulminant necro-haemorrhagic enterocolitis [6]. While clearly a cause of disease in horses, toxigenic strains of *C. difficile* can also be isolated from faeces of healthy horses, with reported isolation rates of 0% to 8% [5,7-9]. While there are numerous point prevalence studies, there has been limited study of colonization of *C. difficile* in different anatomic locations throughout the intestinal tract. *Clostridium difficile* infection (CDI) is typically associated with colonic disease in horses and humans. More recently CDI has also been connected with small intestinal enteritis in humans [10] and with proximal duodenitis jejunitis in horses [11]. This suggests that *C. difficile* is also able to colonize the small intestine.

*Clostridium perfringens* has been associated with enterocolitis in animals, including horses and humans [12,13]. In an earlier study *C. perfringens* type A has been reported to be a normal part of the gastrointestinal flora of healthy horses [14]. Interestingly recent studies have reported low prevalence rates of 0-8% in faeces of healthy adult horses [5,13,15-17]. This organism is mostly associated with colonic disease but has been also identified in the small intestine of horses with gastrointestinal disease [15], in horses with grass sickness and in healthy horses [13]. Nevertheless, clear information about the distribution of *C. perfringens* in the proximal intestinal tract and information on shedding of this organism over time in healthy horses is lacking.

Faecal samples are commonly being used to test for presence *C. difficile* and *C. perfringens;* however, disease does not occur in the distal parts of the gastrointestinal system where the faecal microbiota is established, it occurs in proximal components of the intestinal tract. The physiology and local environments of different intestinal tract locations are quite variable and it is logical that the intestinal microbiota is similarly variable. This is important to study because faecal samples are most often used for diagnosis of intestinal tract disease or for epidemiological studies, yet the degree to which they reflect the status of proximal intestinal tract locations is currently unknown.

Therefore, the objectives of this study were to investigate the presence of *C. difficile* and *C. perfringens* in various intestinal compartments of healthy adult horses and to molecularly characterize isolates.

## Methods

Intestinal contents were collected from 15 euthanized horses greater than one year (Table 1) and free of apparent clinical signs of gastrointestinal disease. The horses were euthanized for reasons unrelated to this study. The legal and ethical requirements of the University of Guelph have been met with regards to the humane treatment of animals described in the study.

**Table 1:** Epidemiologic data of horses included in the study

**Horse**	**#**	**Farm**	**Age in years**	**Gender**	**Breed**	**Reason for euthanasia**
1	#	A	3	MC	Thoroughbred	Cervical vertebral malformation
2		B	14	F	Welsh Pony	Pulmonary calcification
3		C	4	F	Bashkir Curly	EDM
3	#	C	3	F	Bashkir Curly	EDM
5	#	D	3	M	Standardbred	Cervical vertebral malformation
6		C	3	F	Bashkir Curly	EDM
7	#	C	2	M	Bashkir Curly	EDM
8		C	4	F	Bashkir Curly	EDM
9		E	3	F	Standardbred	Cervical vertebral malformation
10	#	C	4	MC	Bashkir Curly	EDM
11	#	F	8	MC	Irish Sport Horse	Fracture L5
12	#	G	3	F	Standardbred	Cervical vertebral malformation
13		H	4	MC	Standardbred	Cervical vertebral malformation
14		I	3	F	Standardbred	Cervical vertebral malformation
15	#	J	6	MC	Hannoverian	Cervical vertebral malformation

# horses from which *C. difficile* was isolated from at least 1 compartment

M = male non castrated, MC = male castrated, F = female, EDM = Equine Degenerative Myeloencephalopathy.

Within four hours after euthanasia samples were collected from the stomach, duodenum, jejunum, ileum, body of the caecum, right dorsal colon, left ventral colon, small colon and rectum. Samples were collected asepetically via enterotomy and intestinal content was collected using sterile forceps and stored at −80°C until processing.

### *Clostridium difficile* culture

Two grams of intestinal content or faeces were inoculated into *C. difficile* moxalactam and norfloxacin broth (CDMN)^a^ and anaerobically incubated at 37°C for five to seven days. An aliquot of the broth was alcohol shocked with an equal volume of anhydrous ethanol for one hour. This mixture was centrifuged for ten minutes at 3800 *g*. The supernatant was discarded and the pellet was streaked onto a CDMN agar plate and anaerobically incubated for 48 hours at 37°C. Suspicious colonies were subcultured onto blood agar, anaerobically incubated for 24 hours at 37°C, and confirmed as *C. difficile* by colony morphology, characteristic odour and production of L-proline aminopeptidase (Pro Disc Remel)^b^.

### DNA extraction

DNA was extracted from a pure culture on blood agar with a Chelex resin-based DNA extraction commercial kit (Insta Gene)^c^, using the manufacturer’s instructions. The resulting DNA was used as template for subsequent PCR tests.

### Ribotyping of *C. difficile*

A previously described ribotyping method was used to analyse all positive isolates [18]. When the ribotype was a recognized international ribotype previously identified by the PHLS Anaerobic Reference Unit (Cardiff, UK), the appropriate numerical designation (i.e. 078) was used. Otherwise, in-house nomenclature was assigned.

### Toxin gene profiling of *C. difficile*

PCR detection of genes encoding toxins A (*tcdA)* and B (*tcdB)* was performed as previously described [19]. The presence of binary toxin (CDT) was tested by amplification of a fragment of the gene (*cdtB)*[20].

### *Clostridium perfringens* culture

Two grams of intestinal content or faeces were inoculated into 8 ml brain heart infusion broth (BHI)^d^ and anaerobically incubated for two days at 37°C. After incubation an aliquot of the broth was alcohol shocked with an equal volume of anhydrous ethanol for one hour and then centrifuged at 3800 *g* for ten minutes. The supernatant was discarded, and the resulting pellet was inoculated onto blood agar and anaerobically incubated at 37°C for 48 hours. *Clostridium perfringens* colonies were identified by colony morphology and double-zone hemolysis. Suspicious colonies were subcultured on blood agar and anaerobically incubated for 48 hours at 37°C. Christie-Atkins-Munch –Peterson (CAMP) testing was performed on colonies accompanied by a positive control (NTC 3110, NTC 8840).

### *Clostridium perfringens* multiplex PCR

Typing of *C. perfringens* was based on genes encoding all four major toxins as well as enterotoxin and β_2_ as described previously [21].

### Statistical analysis

Kappa values were calculated to compare agreement between positive rectum/faecal samples and overall carrier status of *C. difficile*. Agreement was interpreted as follows: poor agreement = kappa less than 0.20, fair agreement = kappa 0.20 to 0.40, moderate agreement = kappa 0.40 to 0.60, good agreement = kappa 0.60 to 0.80, very good agreement = kappa 0.80 to 1.00 [22]. The remaining data were analysed in a descriptive manner.

## Results

### Epidemiologic data of horses and farms

Fifteen horses of more than one year were included in the study (Table 1). These horses originated from ten farms, six horses from a single premise and the remaining nine from different farms. Information on diet, housing and administration of drugs including antimicrobials was not available.

### *Clostridium difficile* prevalence

*Clostridium difficile* was isolated from 14/135 (10.3%) samples from 8/15 (53.3%) horses on 6/10 (60%) farms. Between zero and three/nine (33%) sites were positive per horse, and multiple sites were positive in four (27%) horses (Table 2). Isolates were recovered from the duodenum (n = 1/15, 6.7%), jejunum (n = 1/15, 6.7%), ileum (n = 1/15, 6.7%), right dorsal colon (n = 4/15, 27%), small colon (n = 2/15, 13.3%), and rectum/faeces (n = 5/15, 33%). In three horses (38%) *C. difficile* was isolated from a proximal site but the rectal samples were negative, while in 3 (38%) other horses the rectum and a proximal site were positive and in 2 (25%) horses the rectum was the only positive site. The relative sensitivity of faecal sample culture to detect overall *C: difficile* presence in a horse was 63% (CI 95%:0.29 and 0.89). Agreement between rectal and proximal samples (all proximal sites combined) was good (kappa = 0.61, standard error 0.19).

**Table 2:** **Ribotypes and toxin gene profiles of *Clostridium difficile *isolated from gastrointestinal compartments of healthy horses**

**Horse**	**Farm**	**Compartment**	**Toxin A**	**Toxin B**	**CDT**	**Ribotype**^1^
1	A	Rectum	+	+	+	078
4	C	Right Dorsal Colon	+	+	-	001
		Rectum	+	+	+	078
5	D	Right Dorsal Colon	+	+	-	001
		Rectum	+	+	+	078
7	C	Duodenum	+	+	+	078
		Right Dorsal Colon	+	+	-	001
		Rectum	+	+	+	078
10	C	Small colon	+	+	-	001
11	F	Jejunum	+	+	-	AS2
		Ileum	+	+	-	AS1
		Right Dorsal Colon	+	+	-	AS1
12	G	Rectum	+	+	-	AS1
15	J	Small colon	+	+	-	AS3

^1^ PHLS Anaerobic Reference Unit (Cardiff, UK) when appropriate, otherwise, internal nomenclature was used.

### *Clostridium difficile* toxin detection and ribotyping results

All isolates possessed genes encoding for toxins A and B (Table 2). Five isolates were ribotype 078 and also possessed the gene encoding for part of the binary toxin (*cdtB)*. Four isolates were ribotype 001 and were *cdtB* negative. The remaining five isolates corresponded to three ribotype profiles not previously identified in this laboratory. These five isolates were *tcdA* and *tcdB* positive, but were *cdtB* negative. Interestingly, more than one ribotype was found in all horses from which multiple compartments were positive.

### Prevalence and characterization of *C. perfringens*

*Clostridium perfringens* was only isolated from the left ventral colon of one horse. The detection limit of the method used was low at 9 cfu/g (data not shown). The single isolate contained only the alpha toxin gene, classifying it as *C. perfringens* type A.

## Discussion

This study showed that *C. difficile* can be isolated from a variety of gastrointestinal compartments of healthy horses and that several distinct ribotypes may be isolated from the same horse. Explanations for the different ribotypes in the same horse include simultaneous transient passage of multiple strains through the gastrointestinal system or colonization of different compartments by diverse strains. Regardless, these data indicate that the typical practice of selecting one colony for typing, an almost universal procedure in studies that have reported *C. difficile* types in horses [23,24], might underestimate the true ribotype diversity and distribution. Human studies have reported co-existence of different ribotypes in faecal specimens from healthy humans and those affected with CDI [25,26]; however, the presence of multiple strains is rarely reported. In a study of healthy humans, Kato and co-workers (2001) reported that only 1/94 (1.1%) faecal samples positive for *C. difficile* contained more than one ribotype per sample. Similar data have not been available for horses, but this study suggests that methods able to detect different ribotypes in a single sample might be required to get a true understanding of the ribotype distribution in equine samples. Regardless, the potential presence of multiple *C. difficile* strains in the same animal must be considered when designing and interpreting studies.

*Clostridium difficile* was found in various locations throughout the intestinal tract, which is not surprising given its association with both small and large intestinal disease. As with any study of the intestinal microbiota, it is impossible to definitively state that *C. difficile* was truly colonizing these locations as opposed to passing through transiently following ingestion. This study cannot differentiate between these two, but demonstrates that this organism is adapted to survive and perhaps proliferate in various intestinal locations. Further investigations into the dynamics of *C. difficile* and ways to differentiate transient passage from colonization are required.

Inconsistent detection of *C. difficile* in different intestinal compartments of the same horse could be due to non-homogenous distribution of *C. difficile* in truly colonized animals, intestinal site predilections for *C. difficile* (or specific strains), or presence of *C. difficile* below detection thresholds at some sites.

Overall the prevalence of *C. difficile* in the gastrointestinal tract of horses found in this study (53%) was higher compared to other studies (typically 0-10%), including those performed in the same geographical region [5,7-9]; however, prevalence data should be interpreted with caution, as the study was not designed to determine population prevalence. Also, the presence of multiple animals per farm may have introduced a clustering effect. Six horses in this study originated from one farm, and *C. difficile* was isolated from 3/6 (50%) of them, potentially slightly over estimating overall prevalence data. Given the small sample size and potential for clustering in one farm, this study should not be taken as a population prevalence study, but rather as a comparison among intestinal compartments and assessment of ribotype distribution. The detection of *C. difficile* in feces of healthy horses shows that horses are exposed to this pathogen frequently, however only a small subset of horses eventually develop disease. Better understanding of the epidemiology of this pathogen is required to better prevent and treat disease.

Assessment of faecal samples is the standard practice for diagnosis of disease caused by enteropathogens, because of the ease of access and the impractical nature of sampling other intestinal compartments in most cases. Therefore, understanding how faecal samples reflect proximal compartments is critical for interpretation of the data. Agreement between rectal samples and proximal compartment was good (κ = 0.61), although these data do indicate that rectal samples might not absolutely reflect the status of proximal compartments, both in the presence or absence of *C. difficile* and the ribotypes that are recovered. These findings should be considered when interpreting results from other studies, as prevalence of *C. difficile* in the gastrointestinal tract of horses might be higher than faecal prevalence would suggest.

The presence of a variety of ribotypes in this study is not surprising. Previous studies have reported a wide diversity of ribotypes in horses [24,27]. The most common ribotype in this study was ribotype 078, commonly found in pigs and cattle [28], and increasingly implicated in community-associated CDI in humans [29,30]. While ribotype 078 has been found in horses [9,23,28], the prevalence here is higher compared to previous studies of horses in this region, such as a 2006–2007 surveillance study where ribotype 078 only accounted for 3/52 (5.8%) of isolates from healthy horses [9]. This could indicate that this ribotype is emerging in this region. The second most common strain in this study was ribotype 001. This ribotype has been previously reported in horses [9,27] and is also a predominant human clone, being the most common ribotype in a study of people in hospitals in the region [31]. Overall, nine of the 14 (64%) isolates found in this study were types that have been implicated in CDI in humans in the region [31]. Data such as these raise concern that *C. difficile* may be a zoonotic pathogen [32]; however, proof of interspecies transmission is currently lacking.

The low prevalence of *C. perfringens* in intestinal content samples obtained in this study is consistent with previous studies. While *C. perfringens* is commonly found in horses with enteric disease, foals and pregnant mares, the low prevalence reported here is consistent with other studies of adult horses [13,16]. Methodological issues should always be considered with studies reporting low prevalence. Standard and validated methods are not available for *C. perfringens* isolation from equine faeces; however, the method used here had a detection threshold of 9 cfu/ml of faeces (data not presented) and identical protocols were used in a study that identified *C. perfringens* shedding in 88% of healthy dogs [33]. Thus, it is unlikely that poor test sensitivity accounted for the low prevalence reported here, and these results suggest that *C. perfringens* is uncommonly shed by healthy horses, or shed intermittently or at very low levels. Whether the higher isolation rates from horses with gastrointestinal tract disease indicates cause or simply opportunistic overgrowth of this organism is still unclear [13,15].

A limitation of this study was its small sample size and therefore care has to be taken when interpreting these results; however, this is the first study determining the prevalence and ribotype distribution of *C. difficile* in different compartments of healthy horses and despite the small sample size it gives new insight into the epidemiology of this pathogen.

Information on antimicrobial use in the weeks prior to euthanasia was not available; however, none of the horses had any clinical condition reported that would likely have been treated with parenteral antimicrobials. It is certainly plausible that prior antimicrobial use would have affected results of this study; however, any effect would probably have been on overall prevalence, not an alteration of distribution of *C. difficile* in different intestinal locations. If there were any impact on *C. perfringens,* it would have been expected to increase the prevalence, something that was not observed here given the very low prevalence.

## Conclusions

This study provides new epidemiologic data on the presence and distribution of *C. difficile* and *C. perfringens* throughout the intestinal tract of healthy horses. While faecal samples provide a reasonable indication of the presence or absence of these organisms in the proximal intestinal tract, differences in *C. difficile* distribution and types can be found in different intestinal locations, something that must be considered when designing and interpreting studies relying on faecal sample collection alone. While the gastrointestinal microbiota of the horse remains a poorly understood, complex and important polymicrobial environment, a better understanding of important organisms such as *C. difficile* and *C. perfringens* may help to elucidate the epidemiology and microbiology of the intestinal microbiota in health and disease.

## Endnotes

^a^Oxoid, Nepean Canada.

^b^ProDisc test; Remel, Carr-Scarborough Microbiologicals, Inc, Decatur, GA, USA.

^c^InstaGene Matrix; Bio-Rad, Mississauga, Ontario, Canada.

^d^Oxoid, Nepean, Canada.

## Abbreviations

BHI: Brain heart infusion broth; CAMP: Christie-Atkins-Munch –Peterson testing; CDI: Clostridium difficile infection; CDMN: *C. difficile* moxalactam and norfloxacin broth; CDT: Binary Toxin of Clostridium difficile; DNA: Desoxy-ribonucleic acid; PCR: Polymerase chain reaction.

## Competing interests

The authors declare that they have no competing interests.

## Authors’ contribution

AS was involved in study design, carried out the sample collection, laboratory work and drafted the manuscript. JW conceived the study and was part of the study design and helped with laboratory analysis. LA participated in the design of the study and interpretation of data. HS and participated in its design and coordination and helped to draft the manuscript. PS was involved in study design, coordination and drafting of the manuscript. All authors read and approved the final manuscript.
